# Expression of aquaporin 1, 3 and 5 in colorectal carcinoma: correlation with clinicopathological characteristics and prognosis

**DOI:** 10.3389/pore.2023.1611179

**Published:** 2023-06-02

**Authors:** Guangwen Zhang, Yongfei Hao, Ling Chen, Zengshan Li, Langlang Gao, Jian Tian, Qing Qiao, Jinsong Zhang

**Affiliations:** ^1^ Department of Radiology, Xijing Hospital, Fourth Military Medical University, Xi’an, Shaanxi, China; ^2^ School of Medicine, Yan’an University, Yan’an, Shaanxi, China; ^3^ Department of Pathology, Xijing Hospital, Fourth Military Medical University, Xi’an, Shaanxi, China; ^4^ Department of General Surgery, Tangdu Hospital, Fourth Military Medical University, Xi’an, Shaanxi, China

**Keywords:** biomarker, prognosis, clinical features, colorectal cancer, aquaporin

## Abstract

**Background:** Prognostic biomarkers in colorectal carcinoma (CRC) have an important role in therapeutic strategy. Studies have shown that high expression of Aquaporin (AQP) is associated with poor prognosis in a variety of human tumors. AQP is involved in the initiation and development of CRC. The present study aimed to investigate the correlation between the expression of AQP1, 3 and 5 and clinicopathological features or prognosis in CRC.

**Methods:** The AQP1, 3 and 5 expressions were analyzed based on the immunohistochemical staining of tissue microarray specimens including 112 patients with CRC between June 2006 and November 2008. The expression score of AQP (Allred_score and H_score) was digitally obtained with Qupath software. Patients were divided into high or low expression subgroups based on the optimal cut-off values. The relationship between expression of AQP and clinicopathological characteristics were evaluated using chi-square test, t-test, or one-way ANOVA, when appropriate. Survival analysis of 5-year progression free survival (PFS) and overall survival (OS) was performed with time-dependent ROC, Kaplan-Meier curves, univariate and multivariate COX analysis.

**Results:** The AQP1, 3 and 5 expressions were associated with regional lymph node metastasis, histological grading, and tumor location in CRC, respectively (*p* < 0.05). Kaplan-Meier curves showed that patients with high AQP1 expression had worse 5-year PFS than those with low AQP1 expression (Allred_score: 47% vs. 72%, *p* = 0.015; H_score: 52% vs. 78% *p* = 0.006), as well as 5-year OS (Allred_score: 51% vs. 75%, *p* = 0.005; H_score: 56% vs. 80%, *p* = 0.002). Multivariate Cox regression analysis indicated that AQP1 expression was an independent risk prognostic factor (*p* = 0.033, HR = 2.274, HR95% CI: 1.069–4.836). There was no significant correlation between the expression of AQP3 and 5 and the prognosis.

**Conclusion:** The AQP1, 3 and 5 expressions correlate with different clinicopathological characteristics and the AQP1 expression may be a potential biomarker of prognosis in CRC.

## Introduction

Colorectal carcinoma (CRC) is the second most deadly cancer and the third most prevalent malignant tumor worldwide [1]. In the past few decades, despite aggressive surgery and advances in chemotherapy, the prognosis for CRC remains unsatisfactory, the 5-year survival rate of CRC patients is still less than 64% [2, 3]. Previous studies have shown that the effectiveness of postoperative chemotherapy and patient survival was closely related to tumor biology [4, 5]. Therefore, the discovery of potential biomarkers is imperative to predict the prognosis of CRC.

Aquaporins (AQPs), a family of transmembrane proteins, are widely distributed on the membrane of nucleated cells. The fluid transfer interceded by AQPs is the primary method for water entry into the cells or outside the cells [6]. At present, it is known that 13 kinds of AQPs can be expressed in humans, of which AQP0, AQP1, AQP2, AQP4, AQP5, AQP6, and AQP8 play a major role in selectively transporting water molecules; AQP3, AQP7, AQP9, and AQP10 can transport glycerol and other small molecular solutes [7], while AQP11 and AQP12 are responsible for the transport of H_2_O_2_ in the endoplasmic reticulum [8]. Some clinical studies have indicated that AQPs play an important role in maintaining osmotic pressure, regulating tumor cell migration, cell-matrix adhesion and cell proliferation, and promoting tumor neovascularization [9]. In addition, AQPs have extensive interactions with oncogenes and proteins [10]. Recent research has proved that AQPs were strongly expressed in a variety of human tumors, including, renal cell carcinoma [11], prostate cancer [12], breast cancer [13], pancreatic adenocarcinoma [14], and lung cancer [15].

Although previous studies have confirmed that AQP1 was strongly associated with the prognosis of CRC, a consensus has not been reached until now. Yoshida et al [16] showed that AQP1 was an independent poor prognostic factor for overall survival (OS, *p* = 0.03). On the contrary, Byung et al [17]found that AQP1 was suggested as not an independent prognostic factor for progression free survival (PFS) and OS. Likewise, Imaizumi et al [18] demonstrated that AQP1 expression was not related to the PFS. In our previous research, we found that the ultra-high b-value DWI could match AQP1 expression of tumor and accordingly speculate AQP1 may be a potential biomarker for prognosis [19]. Up until now, few studies have used two expression scores to evaluate the possible correlation between AQP1, 3 and 5 expression and clinicopathological characteristics or prognosis in CRC. Since the AQPs with high expression in CRC are mainly AQP1, 3 and 5 [20], We therefore hope to determine whether AQP1, 3 and 5 could be used as specific biomarkers indicating that CRC has different clinicopathological features and prognosis.

## Materials and methods

### Patients

**FIGURE 1:**
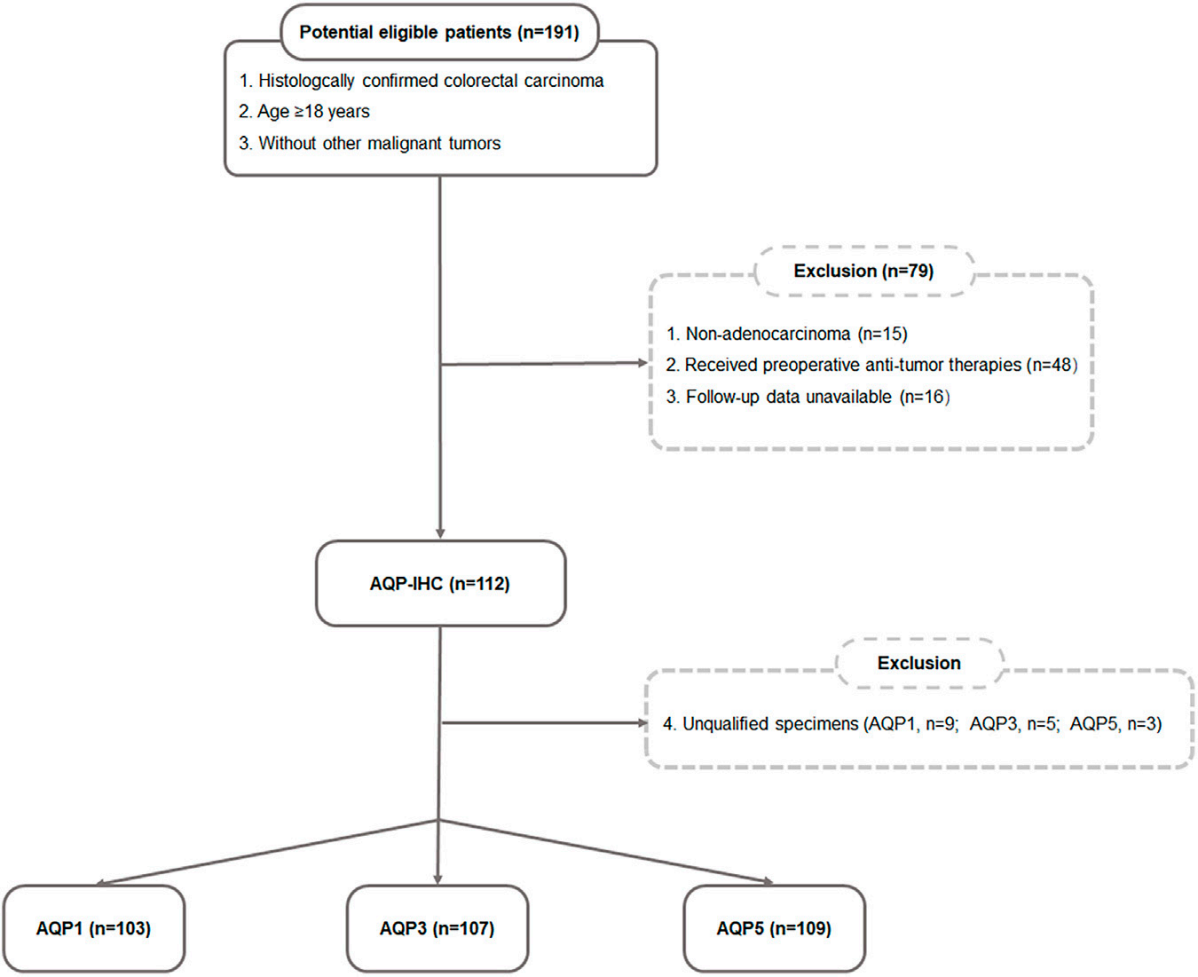
Flow chart of the eligible patient’s selection.

This study was approved by the Ethics Committee of Xijing Hospital and informed consent was acquired for patient specimens and follow-up information. A total of 191 patients with CRC who had undergone surgical resection between June 2006 and November 2008 were investigated and followed up until 2016 in this study. According to the following inclusion and exclusion criteria (Figure 1), the postoperative specimens of 112 patients were involved. The inclusion criteria included: 1) histologically confirmed colorectal adenocarcinoma; 2) age ≥ 18; 3) without other malignant tumors. The exclusion criteria included: 1) non-adenocarcinoma (*n* = 15); 2) received preoperative anti-tumor therapies (*n* = 48); 3) follow-up data unavailable (*n* = 16); 4) unqualified specimens (AQP1, *n* = 9; AQP3, *n* = 5; AQP5, *n* = 3). Lastly, the total number of specimens evaluated for AQP1 expression was 103, AQP3 expression was 107 and AQP5 expression was 109. The main indicators were PFS and OS. PFS was defined as the period of time from the patient’s diagnosis date until any tumor progression, such as metastasis, recurrence, or death. OS was defined as the period of time from the diagnostic date until death from any cause.

### Immunohistochemical staining of AQP1, 3 and 5

Each TMA contained 17 patients’ tissues including 2 cores of tumor tissue and 1 core of paracancerous tissue for one patient. The core diameter of TMA was 1.6 mm. The immunohistochemical staining was performed on a Leica BOND-MAX autoimmunostainer (Leica Instrument Co., Ltd.). The tissue chips were first cut into 4-μm-thick slices using a microtome, attached to slides, dried, and baked at 60°C for 24 h before use. The prepared slices were dewaxed with xylene, rehydrated with different concentrations of ethanol (100% and 95%), rinsed with distilled water, and repaired with heat-induced antigen. Finally, the antigen was detected by an automatic staining machine (Benchmark) and Bond Polymer Purification Detection Kit (LeicaInstrumentCo., Ltd.). The primary antibodies (Abcam) were diluted as follows: anti-AQP1 (ab168387, 1:1,000), anti-AQP3 (ab125219, 1:500), and anti-AQP5 (ab92320, 1:200).

### Scoring of AQP immunohistochemical staining

All slides were scanned with a Nano Zoomer SQ (HAMAMATSU, Japan). The expression levels of AQP1, 3 and 5 in the tumor region of each core of TMA were evaluated by Qupath software (Quantitative Pathology, version 0.2.0) and corresponding scores (Allred_score [21]and H_score [22]) were obtained. The average score of two tumor cores from the same patient was submitted for statistical analysis.

The combination of the values for staining intensity and the percentage of positively stained cells produced an Allred score that ranged from 0 to 8. The intensity was given a score between 0 and 3, with 0 representing no staining, 1 representing weak staining, 2 representing moderate staining, and 3 representing high staining Scores representing the proportion of positively stained tumor cells were stratified as 0 (no positive tumor cells), 1 (<1%), 2 (1%–10%), 3 (10%–33%), 4 (33%–67%) and 5 (67%–100%).

The H_score which ranged from 0 to 300 was calculated with the following equation: H_score = ∑ (*PI*  × * I*) = (percentage of cells with weak intensity  ×  1) + (percentage of cells with moderate intensity ×  2) + (percentage of cells with strong intensity × 3). The terms *PI* and *I* in the formula represent the proportion of positive cells to all cells and the staining intensity (0–3), respectively.

### Statistical analysis

All analyses were completed using the statistical programming language R (version 4.1.2, 6.1, www.r-project.org) and SPSS (version 26.0, IBM, Armonk, NY). For all statistical tests, differences were considered statistically significant when the *p*-value was <0.05 (two-tailed).

The correlations between AQP expression and clinicopathological characteristics were investigated with chi-square test, *t*-test, or one-way ANOVA, when appropriate. The optimal cut-off values of AQP (Allred_score and H_score) to distinguish high AQP1, 3 and 5 expressions from low expression were determined separately using time-dependent ROC curves (R package “survival ROC”). The Kaplan-Meier curves and log-rank tests were used to perform survival analysis of AQP expression by R package “survival.” The Cox proportional hazard regression models were used to conduct both univariate and multivariate survival analyses.

## Results

### Patient characteristics

A total of 112 patients were eventually enrolled in this retrospective study. The AQP1 group contained 103 consecutive patients (66 males, 37 females; mean age, 59.5 ± 13.4 years; mean follow-up, 78 months, range: 1–128 months). The AQP3 group contained 107 consecutive patients (67 males, 40 females; mean age, 58.8 ± 14.0 years; mean follow-up, 79 months, range: 1–128 months). The AQP5 group contained 109 consecutive patients (68 males, 41 females; mean age, 58.9 ± 14.0 years; mean follow-up, 78 months, range: 1–128 months). The other clinical characteristics of patients were presented in Table 1.

**TABLE 1:** Clinicopathological characteristics of patients in AQP1, 3 and 5 groups.

Characteristics	AQP1 (*n* = 103)	AQP3 (*n* = 107)	AQP5 (*n* = 109)
Age, years (mean ± SD), n (%)	59.5 ± 13.4	58.8 ± 14.0	58.9 ± 14.0
<60	50 (48.5%)	53 (49.5%)	54 (49.5%)
≥60	53 (51.5%)	54 (50.5%)	55 (50.5%)
Gender, n (%)			
Male	66 (64.1%)	67 (62.6%)	68 (62.4%)
Female	37 (35.9%)	40 (37.4%)	41 (37.6%)
Primary tumor site, n (%)			
Left colon	61 (59.2%)	60 (56.1%)	62 (56.9%)
Right colon	42 (40.8%)	47 (43.9%)	47 (43.1%)
Histological grade, n (%)			
Well differentiated	33 (32.0%)	35 (32.7%)	36 (33.0%)
Moderate differentiated	62 (60.2%)	61 (57.0%)	62 (56.9%)
Poor differentiated	8 (7.8%)	11 (10.3%)	11 (10.1%)
TNM stage, n (%)			
I	22 (21.4%)	24 (22.4%)	24 (22.0%)
II	42 (40.8%)	40 (37.4%)	42 (38.6%)
III	22 (21.4%)	25 (23.4%)	25 (22.9%)
IV	17 (16.5%)	18 (16.8%)	18 (16.5%)
T stage, n (%)			
T2	25 (24.3%)	27 (25.2%)	27 (24.8%)
T3	26 (25.2%)	27 (25.2%)	28 (25.7%)
T4	52 (50.5%)	53 (49.5%)	54 (49.5%)
N stage, n (%)			
N−	69 (67%)	70 (65.4%)	72 (66.1%)
N+	34 (33%)	37 (34.6%)	37 (33.9%)
M stage, n (%)			
M−	86 (83.5%)	89 (83.2%)	91 (83.5%)
M+	17 (16.5%)	18 (16.8%)	18 (16.5%)
Treatment strategy, n (%)			
Surgery only	31 (30.1%)	33 (30.8%)	34 (31.2%)
Surgery + other treatment	72 (69.9%)	74 (69.2%)	75 (68.8%)
Survival rate, n (%)			
3-year PFS	68%	68%	67%
3-year OS	70%	70%	69%
5-year PFS	60%	60%	59%
5-year OS	61%	62%	61%

Abbreviations: PFS, progression free survival; OS, overall survival. The pathological stage was determined by the pathologist according to the 7th edition of AJCC.

**FIGURE 2:**
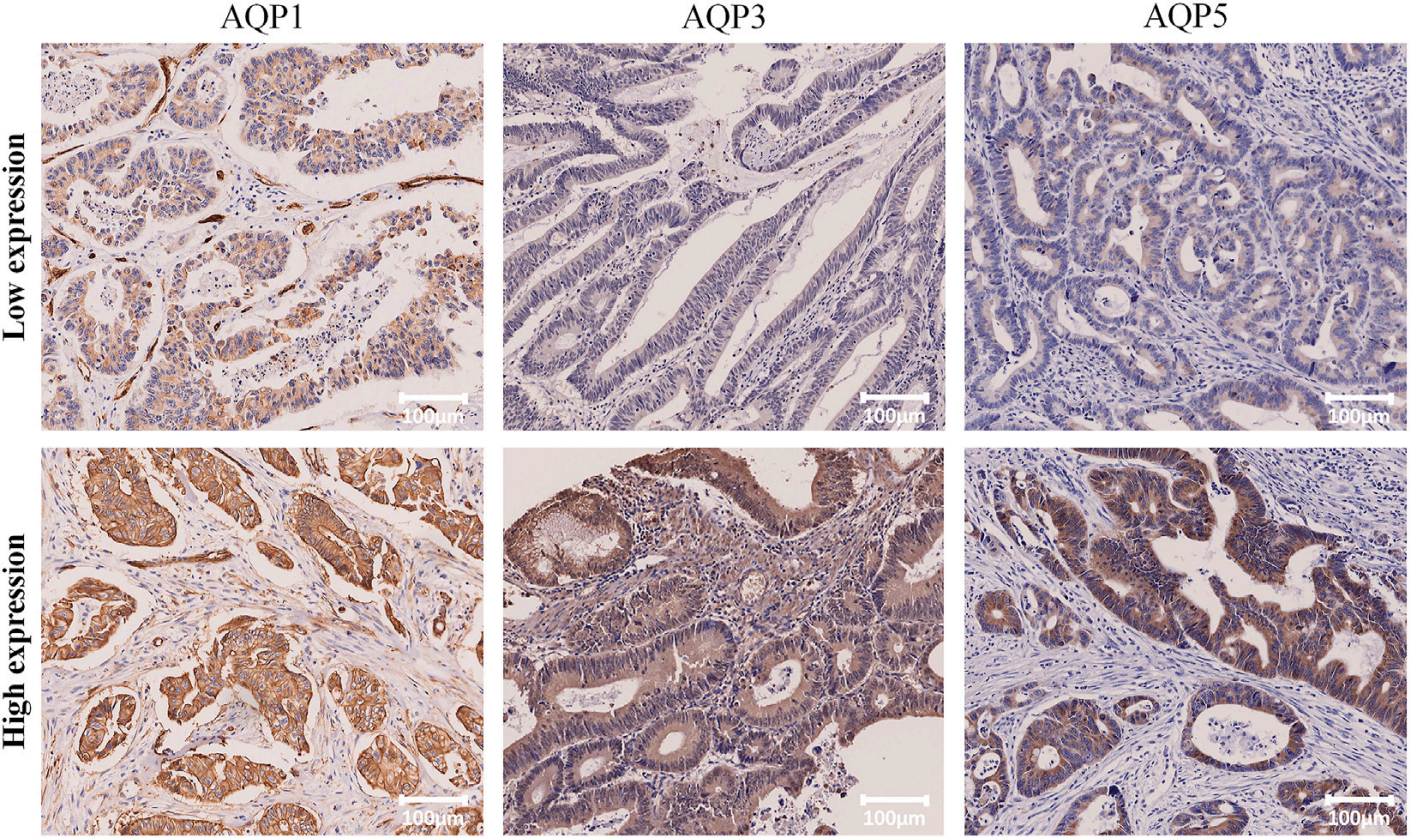
Representative IHC staining of high and low expression of AQP1, AQP3 and AQP5 (×100).

Patients were divided into high or low expression subgroups according to the optimal cut-off values (Allred_score and H_score) of AQP1, 3 and 5 expressions which were determined by time-dependent ROC curves according to the 5-year PFS. The optimal cut-off values for AQP1 (Allred_score) and AQP1 (H_score) were 6.0 and 116.17, respectively. The optimal cut-off values for AQP3 (Allred_score) and AQP3 (H_score) were 5.5 and 115.64, respectively. The optimal cut-off values for AQP5 (Allred_score) and AQP5 (H_score) were 6.0 and 18.64, respectively. Accordingly, Immunohistochemical examples of low and high expression of AQP1, 3 and 5 were shown in Figure 2.

### Associations between AQP1, 3 and 5 expressions and clinicopathological characteristics

**FIGURE 3:**
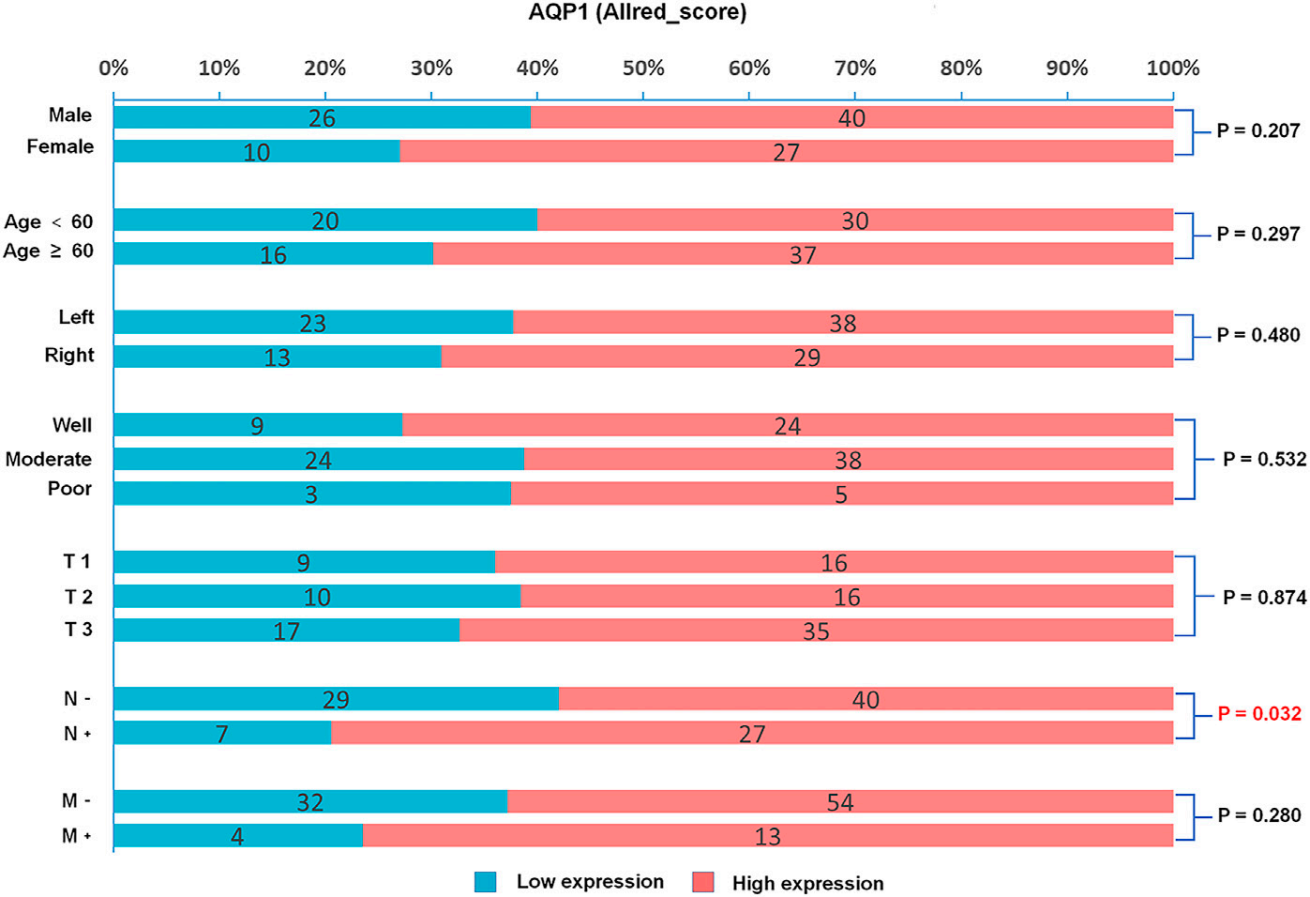
Percentage bar graph about the correlation between AQP1 (Allred_score) expression and clinicopathological characteristics. The AQP1 expression was significantly correlated with the N stage of CRC (χ^2^ = 4.605, *p* = 0.032).

**FIGURE 4:**
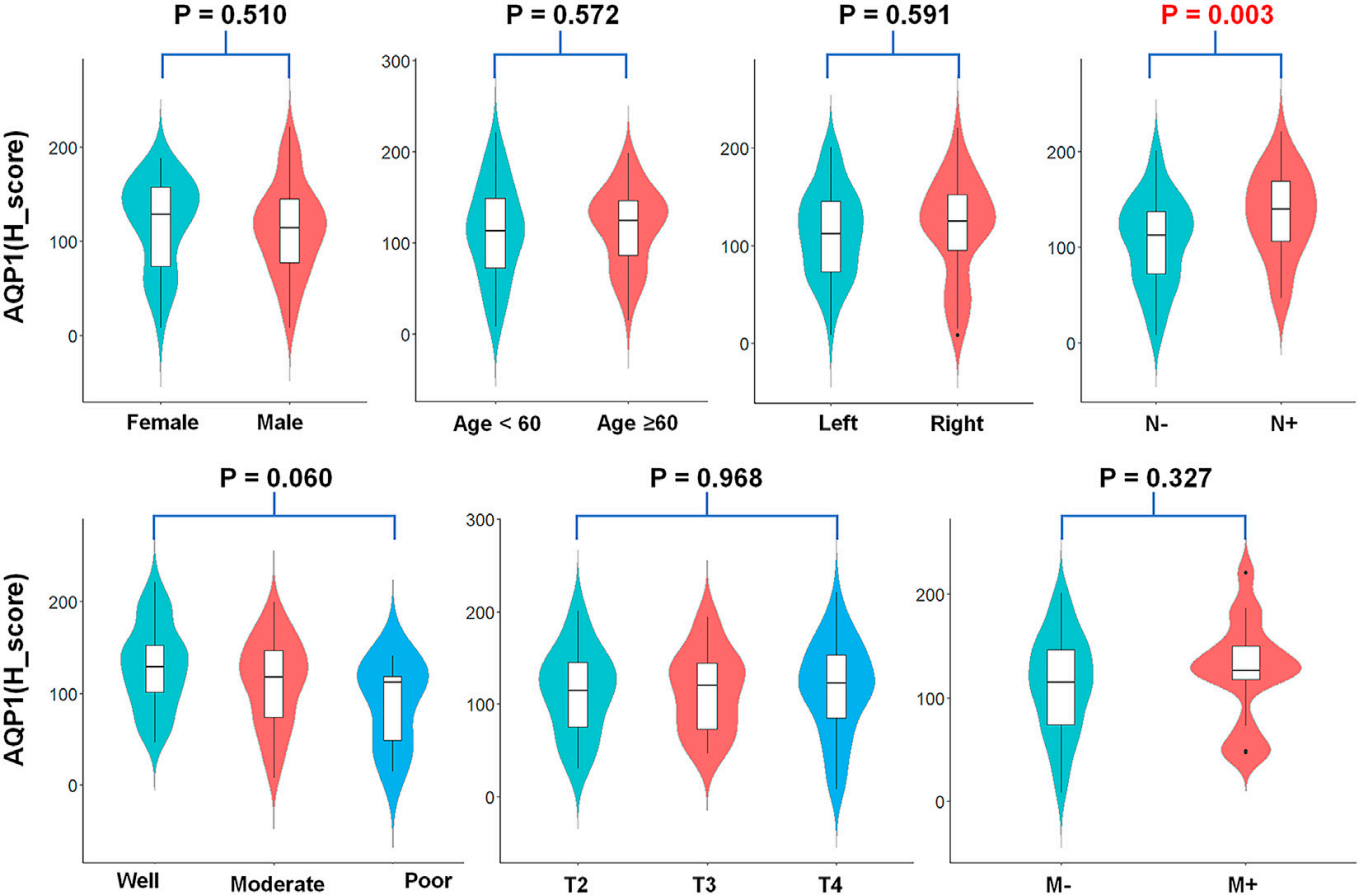
Violin plots about AQP1 (H_score) expression and clinicopathological characteristics. The AQP1 (H_score) expression was significantly correlated with the N stage of CRC (*t* = 3.016, *p* = 0.003).

This study investigated the relationship between AQP1, 3 and 5 expression and gender, age, primary tumor site, histological grading, and TNM stage. For Allred_score, the rate of AQP1 high expression in the positive lymph node group was significantly more common than that in the negative lymph node group (79.4% vs. 58.0%, χ^2^ = 4.605, *p* = 0.032, Figure 3). For H_score, the expression of AQP1 was significantly higher in positive lymph node group than that in negative lymph node group (134.81 ± 44.573 vs. 105.978 ± 46.122, *t* = 3.016, *p* = 0.003, Figure 4).

The AQP3(Allred_score) was significantly correlated with the histological grading of CRC. (χ^2^ = 10.773, *p* = 0.005, Supplementary Figure S1), AQP3 (H_score) had a similar conclusion (*F* = 4.212, *p* = 0.017, Supplementary Figure S2). In multiple comparisons between groups, AQP3 expression (H_score) was found to be significantly higher in well-differentiated group than in moderate-differentiated group (*p* = 0.005, Supplementary Figure S2), while no statistically significant differences in AQP3 expression were found between the well-differentiated group and poor-differentiated group, as well as moderate-differentiated group and poor-differentiated group (*p* = 0.233 and 0.542, respectively).

The AQP5 (Allred_score) was apparently associated with the tumor location of colorectal carcinoma (χ^2^ = 8.123, *p* = 0.004, Supplementary Figure S3), AQP5 (H_score) had a similar conclusion, resulting in significantly lower AQP5 expression in the left hemicolectomy than in the right hemicolectomy (50.473 ± 54.175 vs. 90.052 ± 62.624, *t* = −3.531, *p* = 0.001, Supplementary Figure S4).

### Kaplan-Meier survival curves with AQP1, 3 and 5 expressions

**FIGURE 5:**
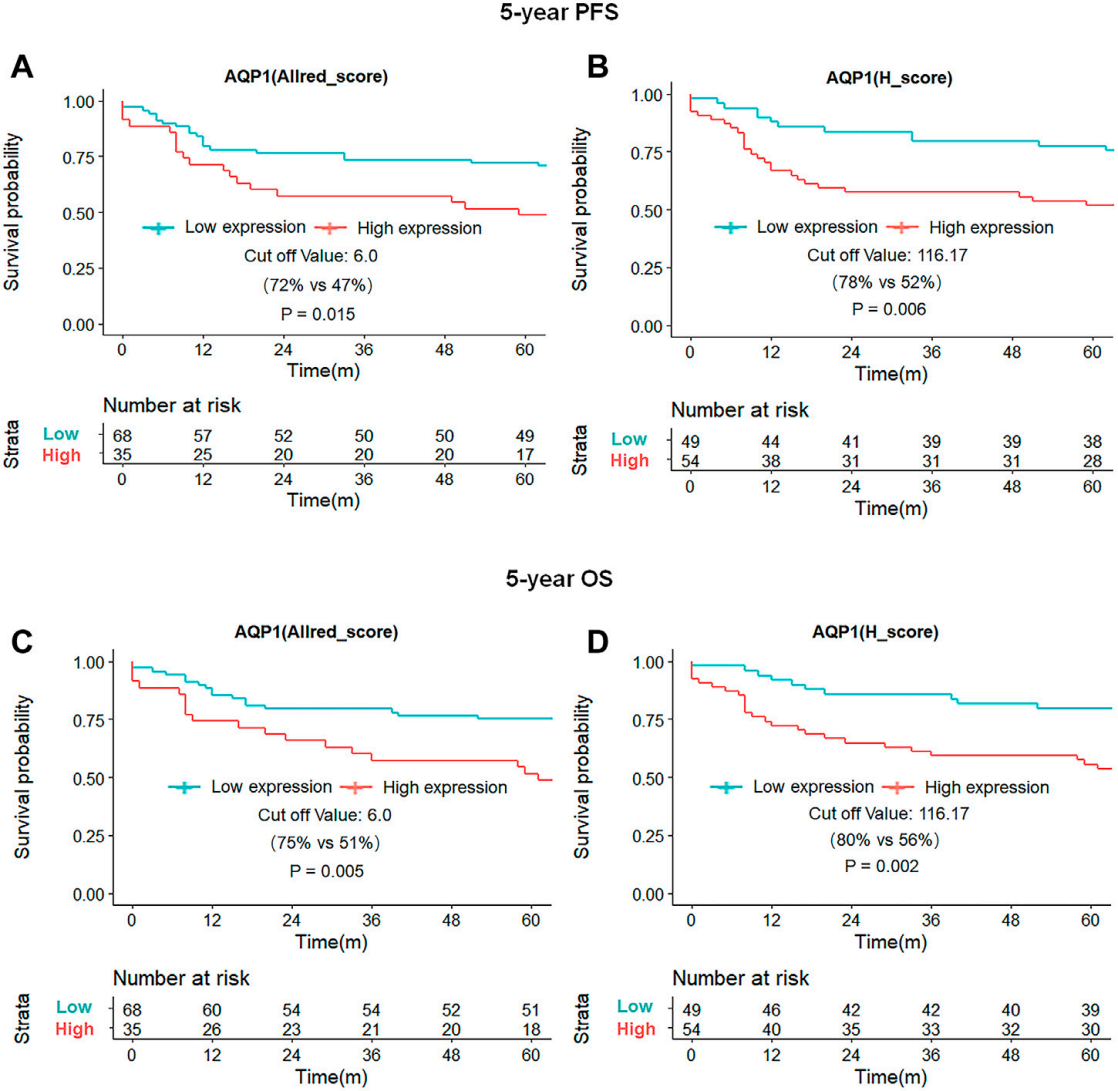
Kaplan-Meier survival curves of 5-year PFS and OS for patients based on AQP1 expression. Patients with high AQP (Allred_score) expression had worse 5-year PFS and OS than those with low AQP (Allred_score) expression [47% vs. 72%, *p* = 0.015, **(A)**; 51% vs. 75%, *p* = 0.005; **(C)**], with similar results for AQP1 (H_score) expression [52% vs. 78%, *p* = 0.006, **(B)**; 56% vs. 80%, *p* = 0.002; **(D)**].

Kaplan-Meier survival curves demonstrated that the patient with high expression of the AQP1 had poorer 5-year PFS than patients with low expression of the AQP1 (Allred_score, 5-year PFS = 47% vs. 72%, *p* = 0.015, Figure 5A; H_score, 5-year PFS = 52% vs. 78%, *p* = 0.006; Figure 5B). Likewise, the survival rate of patients with high AQP1 expression in 5-year OS was significantly lower compared to that of patients with low AQP1 expression in 5-year OS (Allred_score, 5-year OS = 51% vs. 75%, *p* = 0.005, Figure 5C; H_score, 5-year OS = 56% vs. 80%, *p* = 0.002; Figure 5D). However, Kaplan-Meier survival curves presented that the 5-year PFS and OS of patients in high or low AQP3 and 5 expression groups had no statistical difference. (*p* > 0.05, Supplementary Figures S5, S6).

### Univariate and multivariate COX regression analysis of the prognosis

The AQP1, 3 and 5 expression and clinicopathologic factors, including age, gender, primary tumor site, histological grading, TNM-stage and treatment strategy, were submitted into univariate and multivariate COX regression analysis. The univariate Cox regression analysis showed that AQP1 (Allred_score and H_score), age, and TNM-stage were risk factors of 5-year PFS and OS for CRC (*p* < 0.05), while the expression of AQP 3 and AQP5 were not correlated with 5-year PFS and OS (*p* > 0.05, Table 2).

**TABLE 2:** Univariate cox regression analysis for prognosis of colorectal carcinoma.

Variate	PFS		OS	
	*P*	HR (95% CI)	*P*	HR (95% CI)
AQP1 (Allred_score ≥6)	**0.017**	2.144 (1.143–4.021)	**0.007**	2.433 (1.276–4.641)
AQP3 (Allred_score ≥5.5)	0.588	1.187 (0.638–2.211)	0.405	1.319 (0.688–2.528)
AQP5 (Allred_score ≥6)	0.349	1.402 (0.691–2.845)	0.897	1.043 (0.550–1.979)
AQP1 (H_score ≥116.17)	**0.008**	2.532 (1.282–5.004)	**0.003**	3.041 (1.471–6.288)
AQP3 (H_score ≥115.64)	0.458	1.283 (0.664–2.478)	0.495	1.248 (0.660–2.361)
AQP5 (H_score ≥18.64)	0.368	1.385 (0.682–2.809)	0.521	1.264 (0.618–2.586)
Gender (male)	0.656	0.859 (0.442–0.163)	0.898	0.957 (0.487–1.880)
Age (≥60)	**0.042**	1.971 (1.024–3.794)	**0.030**	2.115 (1.076–4.157)
Primary tumor site (left)	0.969	0.988 (0.522–1.870)	0.673	0.869 (0.454–1.666)
Histological grade (low)	0.475	1.219 (0.708–2.100)	0.213	1.428 (0.815–2.501)
TNM-stage (III–IV)	**<0.001**	3.084 (1.624–5.858)	**<0.001**	3.122 (1.614–6.038)
Treatment (comprehensive)	0.064	0.544 (0.287–1.037)	0.130	0.598 (0.308–1.163)

Abbreviations: PFS, progression-free survival; OS, overall survival; HR (95% CI), hazard ratio and its 95% confidence interval. Univariate and multivariate COX regression analyses of clinicopathological characteristics were performed based on the data of the AQP1 group. The score of AQP expression (<cut-off value), age (<60 years old), gender (female), primary tumor site (right), histological grade (high and moderate), TNM-stage (I–II) and treatment modality (surgery only) were set as references in COX regression analyses. Comprehensive treatment indicated that patients received surgery plus with neoadjuvant therapy and or adjuvant therapy.

Bold values are significant at *p* < 0.05.

Since the Allred_score and H_score were two indicators to evaluate the same protein expression and the univariate COX analysis showed that the H_score was more significant than Allred_score in PFS and OS assessment (PFS, *p* = 0.008 vs. 0.017; OS, *p* = 0.003 vs. 0.008), the multivariate COX regression analysis was performed using H_score of AQP1 and indicated that AQP1 was no longer an independent risk factor for 5-year PFS (*p* = 0.086, HR = 1.866, HR95% CI: 0.916–3.800), but an independent risk factor for 5-year OS (*p* = 0.033, HR = 2.274, HR95% CI: 1.069–4.836, Table 3).

**TABLE 3:** Multivariate cox regression analysis for prognosis of colorectal carcinoma.

Variate	PFS		OS	
	*P*	HR95% CI	*P*	HR95% CI
Age (≥60 years)	0.252	1.483 (0.756–2.911)	0.229	1.533 (0.764–3.076)
TNM stage (III-IV)	**0.007**	2.494 (1.285–4.839)	**0.010**	2.450 (1.243–4.827)
AQP1 (H_score ≥116.17)	0.086	1.866 (0.916–3.800)	**0.033**	2.274 (1.069–4.836)

Abbreviations: PFS, progression free survival; OS, overall survival; HR (95% CI), 95% confidence interval of the hazard ratio. Multivariate COX regression analysis of clinicopathological characteristics was conducted using AQP1 group data. The age (<60 years), TNM stage (I–II) and AQP1 (H_score <116.17) were set as references in COX regression analyses.

Bold values are significant at *p* < 0.05.

## Discussion

In the present study, we investigated the correlation between AQP1, AQP3 and AQP5 expression and clinicopathological features or prognosis in 121 patients with CRC. The AQP1 expression, AQP3 expression and AQP5 expression were related to regional lymph node metastasis, histological grading, and tumor location of CRC, respectively. In survival analysis, the AQP1 high expression was related to poor 5-year PFS and OS and was an independent prognostic factor for 5-year OS, but the AQP3 and AQP5 expression were not significantly correlated with prognosis in CRC.

This study found that AQP1 (H_score) expression was significantly higher in the positive lymph node group than negative lymph node group (134.81 ± 44.573 vs. 105.978 ± 46.122, *p* = 0.003) and AQP1 (H_score) high expression group had significantly worse 5-year PFS than that of the AQP1 low expression group (52% vs. 78%, *p* = 0.006). These results were similar to the work by Yoshida et al. [16] who found that high AQP1 expression in stage II/III colon cancer was strongly associated with regional lymph node metastasis and the 5-year survival rate was significantly lower in the positive AQP1 expression group than the negative group (73.7% vs. 87.9%, *p* = 0.03). In contrast, Byung et al. [17] showed that patients with AQP1 high expression are less likely to develop regional lymph node metastasis which was inconsistent with the theory that high AQP1 expression can promote tumor invasion and metastasis.

Although the molecular mechanism of AQP1 in cancer biology is not fully understood, numerous studies have indicated that AQP1 is involved in the angiogenesis, migration, and proliferation of tumor cells, which plays an important role in the development of cancer [23]. Potential downstream effectors in the signaling pathways involved in AQP1-mediated tumor progression include β-linked protein, Lin-7, FAK, MMP2, MMP9, and histone proteinase B [24]. A representative study reported that AQP1 may enhance the migratory ability and invasiveness of tumor cells by mediating the Wnt/β-catenin signaling pathway and stabilizing the cadherin/β-catenin/Lin-7/F-actin complex on cell membranes [25, 26]. β-catenin as an intracellular signal transducer in the canonical Wnt signaling pathway regulates the expression of downstream genes (c-Myc, cyclinD1, c-Jun, and FRA1), which leads to cell proliferation, differentiation, and inhibition of apoptosis [27]. Therefore, regional lymph node metastasis should be associated with high AQP1 expression, rather than low expression. The high AQP1 expression was strongly associated with worse 5-year PFS and OS, which supports that high AQP1 expression can promote tumor invasion and reduce patient survival. There was no significant correlation between AQP1 expression and other clinicopathological characteristics (gender, age, tumor location, histological grading, T stage, and M stage).

The high expression of AQP3 has been observed in several cancers, which promotes to metastasis, proliferation, and epithelial-to-mesenchymal transition (EMT) [28]. Despite previous studies found that the high expression of AQP3 was related to the degree of lymphatic metastasis and differentiation of CRC [29], this study only found that the expression of AQP3 was related to the histological grading of CRC. Specifically, there was no linear relationship between AQP3 expression and histological grading which may be partially attributed to the small sample size of patients with low differentiation (*n* = 11). Further studies with more low differentiation of sample sizes are needed to validate the result.

In a recent study, Byung et al. [17] showed that AQP5 was not associated with PFS and OS of CRC. This outcome is contrary to that of Tao Shan et al. [30] who found that AQP5 was closely correlated with poor prognosis. According to our current study, the expression of AQP5 was related to the location of the tumor which might remind us that AQP5 played a different role in left and right-sided CRC. There was no significant correlation between AQP5 expression and other clinicopathological characteristics (age, gender, histological grading, and TNM stage).

Although both Allred_score and H_score are semi-quantitative indicators for evaluating protein expression, the range of H_score (0–300) is significantly higher than that of Allred_score (0–8). Therefore, H_score can be analyzed as a continuous variate. As the statistical efficiency of the H_score was generally better than Allred_score in this study, it was considered that H_score could describe AQP expression more accurately than Allred_score and was a better indicator for evaluating protein immunohistochemical results. Qupath is a free open-source digital pathological analysis software developed by the Center for Cancer Research and Cell Biology of Queen’s University in Belfast, which has been widely used all over the world [31]. Several studies published in Nature [32], Cell [33], and Science [34] have demonstrated that quantitative analysis of biomarkers using Qupath software has good reliability and repeatability.

This study has some potential limitations. Firstly, it had a retrospective nature and a relatively small number of patients. In particular, there were only 11 cases of poor-differentiated CRC, more subjects need to be included in further evaluation. Secondly, the heterogeneity of staining may be affected by different sampling methods (TMA vs. whole slides), which is a possible reason that may contribute to different results in the literature. In this study, two tissue cores were carefully selected by a pathologist with 16-year experiences in gastrointestinal cancer, which may compensate for the insufficient representativity of TMA to some extent. Furthermore, we performed a long-time follow-up and digital scoring method for IHC staining, which may supply solid support for the conclusion of this study.

In conclusion, our data suggested that the expression levels of AQP1, AQP3 and AQP5 were associated with regional lymph node metastasis, histological grading, and tumor location of CRC, respectively. However, only AQP1 expression level was an independent risk factor for the prognosis of CRC.

## Data Availability

The original contributions presented in the study are included in the article/Supplementary Material, further inquiries can be directed to the corresponding authors.

## References

[1] Sung H Ferlay J Siegel RL Laversanne M Soerjomataram I Jemal A Global cancer statistics 2020: GLOBOCAN estimates of incidence and mortality worldwide for 36 cancers in 185 countries. CA Cancer J Clin (2021) 71:209–49. 10.3322/caac.21660 33538338

[2] Bender U Rho YS Barrera I Aghajanyan S Acoba J Kavan P. Adjuvant therapy for stages II and III colon cancer: Risk stratification, treatment duration, and future directions. Curr Oncol (2019) 26:S43–s52. 10.3747/co.26.5605 31819709 PMC6878933

[3] Siegel RL Miller KD Sauer AG Fedewa SA Butterly LF Anderson JC Colorectal cancer statistics, 2020. Ca-a Cancer J Clinicians (2020) 70:145–64. 10.3322/caac.21601 32133645

[4] Malki A Abu ElRuz R Gupta I Allouch A Vranic S Al Moustafa AE. Molecular mechanisms of colon cancer progression and metastasis: Recent insights and advancements. Int J Mol Sci (2021) 22:130. 10.3390/ijms22010130 PMC7794761 33374459

[5] Bregni G Telli TA Camera S Deleporte A Moretti L Bali AM Adjuvant chemotherapy for rectal cancer: Current evidence and recommendations for clinical practice. Cancer Treat Rev (2020) 83:101948. 10.1016/j.ctrv.2019.101948 31955069

[6] Ribatti D Ranieri G Annese T Nico B. Aquaporins in cancer. Biochim Biophys Acta (2014) 1840:1550–3. 10.1016/j.bbagen.2013.09.025 24064112

[7] Zannetti A Benga G Brunetti A Napolitano F Avallone L Pelagalli A. Role of aquaporins in the physiological functions of mesenchymal stem cells. Cells (2020) 9:2678. 10.3390/cells9122678 33322145 PMC7763964

[8] Prata C Hrelia S Fiorentini D. Peroxiporins in cancer. Int J Mol Sci (2019) 20:1371. 10.3390/ijms20061371 30893772 PMC6471688

[9] Moon CS Moon D Kang SK. Aquaporins in cancer biology. Front Oncol (2022) 12:782829. 10.3389/fonc.2022.782829 35847914 PMC9278817

[10] Papadopoulos MC Saadoun S. Key roles of aquaporins in tumor biology. Biochim Biophys Acta-Biomembranes (2015) 1848:2576–83. 10.1016/j.bbamem.2014.09.001 25204262

[11] Morrissey JJ Mellnick VM Luo J Siegel MJ Figenshau RS Bhayani S Evaluation of urine aquaporin-1 and perilipin-2 concentrations as biomarkers to screen for renal cell carcinoma A prospective cohort study. Jama Oncol (2015) 1:204–12. 10.1001/jamaoncol.2015.0213 26181025 PMC4617549

[12] Kushwaha PP Verma S Gupta S. Aquaporins as prognostic biomarker in prostate cancer. Cancers (Basel) (2023) 15:331. 10.3390/cancers15020331 36672280 PMC9856769

[13] Rodrigues C Milkovic L Bujak IT Tomljanovic M Soveral G Cipak Gasparovic A. Lipid profile and aquaporin expression under oxidative stress in breast cancer cells of different malignancies. Oxid Med Cell Longev (2019) 2019:2061830. 10.1155/2019/2061830 31379986 PMC6657669

[14] Chen G Song H Yang Z Du T Zheng Y Lu Z AQP5 is a novel prognostic biomarker in pancreatic adenocarcinoma. Front Oncol (2022) 12:890193. 10.3389/fonc.2022.890193 35619903 PMC9128544

[15] Kao YC Jheng JR Pan HJ Liao WY Lee CH Kuo PL. Elevated hydrostatic pressure enhances the motility and enlarges the size of the lung cancer cells through aquaporin upregulation mediated by caveolin-1 and ERK1/2 signaling. Oncogene (2017) 36:863–74. 10.1038/onc.2016.255 27499095

[16] Yoshida T Hojo S Sekine S Sawada S Okumura T Nagata T Expression of aquaporin-1 is a poor prognostic factor for stage II and III colon cancer. Mol Clin Oncol (2013) 1:953–8. 10.3892/mco.2013.165 24649276 PMC3916155

[17] Kang BW Kim JG Lee SJ Chae YS Jeong JY Yoon GS Expression of aquaporin-1, aquaporin-3, and aquaporin-5 correlates with nodal metastasis in colon cancer. Oncology (2015) 88:369–76. 10.1159/000369073 25721378

[18] Imaizumi H Ishibashi K Takenoshita S Ishida H. Aquaporin 1 expression is associated with response to adjuvant chemotherapy in stage II and III colorectal cancer. Oncol Lett (2018) 15:6450–6. 10.3892/ol.2018.8170 29725400 PMC5920209

[19] Zhang G Ma W Dong H Shu J Hou W Guo Y Based on histogram analysis: ADC(aqp) derived from ultra-high b-value DWI could be a non-invasive specific biomarker for rectal cancer prognosis. Sci Rep (2020) 10:10158. 10.1038/s41598-020-67263-4 32576929 PMC7311405

[20] Moosavi MS Elham Y. Aquaporins 1, 3 and 5 in different tumors, their expression, prognosis value and role as new therapeutic targets. Pathol Oncol Res (2020) 26:615–25. 10.1007/s12253-019-00646-9 30927206

[21] Harvey JM Clark GM Osborne CK Allred DC. Estrogen receptor status by immunohistochemistry is superior to the ligand-binding assay for predicting response to adjuvant endocrine therapy in breast cancer. J Clin Oncol (1999) 17:1474–81. 10.1200/jco.1999.17.5.1474 10334533

[22] McCarty KS Jr Miller LS Cox EB Konrath J McCarty KS. Estrogen receptor analyses. Correlation of biochemical and immunohistochemical methods using monoclonal antireceptor antibodies. Arch Pathol Lab Med (1985) 109:716–21. 3893381

[23] Ribatti D Ranieri G Annese T Nico B. Aquaporins in cancer. Biochim Biophys Acta-General Subjects (2014) 1840:1550–3. 10.1016/j.bbagen.2013.09.025 24064112

[24] Tomita Y Dorward H Yool AJ Smith E Townsend AR Price TJ Role of aquaporin 1 signalling in cancer development and progression. Int J Mol Sci (2017) 18:299. 10.3390/ijms18020299 28146084 PMC5343835

[25] Meng FB Rui YF Xu LL Wan C Jiang XH Li G. Aqp1 enhances migration of bone marrow mesenchymal stem cells through regulation of FAK and beta-catenin. Stem Cell Development (2014) 23:66–75. 10.1089/scd.2013.0185 PMC3870604 23962074

[26] Clevers H Nusse R. Wnt/β-catenin signaling and disease. Cell (2012) 149:1192–205. 10.1016/j.cell.2012.05.012 22682243

[27] Tetsu O McCormick F. Beta-catenin regulates expression of cyclin D1 in colon carcinoma cells. Nature (1999) 398:422–6. 10.1038/18884 10201372

[28] Marlar S Jensen HH Login FH Nejsum LN. Aquaporin-3 in cancer. Int J Mol Sci (2017) 18:2106. 10.3390/ijms18102106 28991174 PMC5666788

[29] Li A Lu DH Zhang YP Li J Fang Y Li F Critical role of aquaporin-3 in epidermal growth factor-induced migration of colorectal carcinoma cells and its clinical significance. Oncol Rep (2013) 29:535–40. 10.3892/or.2012.2144 23165320

[30] Shan T Cui X Li W Lin W Li Y. AQP5: A novel biomarker that predicts poor clinical outcome in colorectal cancer. Oncol Rep (2014) 32:1564–70. 10.3892/or.2014.3377 25109507

[31] Humphries MP Maxwell P Salto-Tellez M, QuPath: The global impact of an open source digital pathology system. Comput Struct Biotechnol J (2021) 19:852–9. 10.1016/j.csbj.2021.01.022 33598100 PMC7851421

[32] Roberti MP Yonekura S Duong CPM Picard M Ferrere G Alou MT Chemotherapy-induced ileal crypt apoptosis and the ileal microbiome shape immunosurveillance and prognosis of proximal colon cancer. Nat Med (2020) 26:919–31. 10.1038/s41591-020-0882-8 32451498

[33] Choudhury SR Babes L Rahn JJ Ahn BY Goring KAR King JC Dipeptidase-1 is an adhesion receptor for neutrophil recruitment in lungs and liver. Cell (2019) 178:1205–21. 10.1016/j.cell.2019.07.017 31442408

[34] Nejman D Livyatan I Fuks G Gavert N Zwang Y Geller LT The human tumor microbiome is composed of tumor type-specific intracellular bacteria. Science (2020) 368:973–80. 10.1126/science.aay9189 32467386 PMC7757858

