# Supplementary material for: Expression of aquaporin 1, 3 and 5 in colorectal carcinoma: correlation with clinicopathological characteristics and prognosis
Source: Pathol Oncol Res. 2023 Jun 2;29:1611179. doi: 10.3389/pore.2023.1611179 (PMC10272351; doi:10.3389/pore.2023.1611179)
Supplement: Supplementary file 1 [file DataSheet1.PDF]

**Supplementary Material**

**Supplementary Figure**

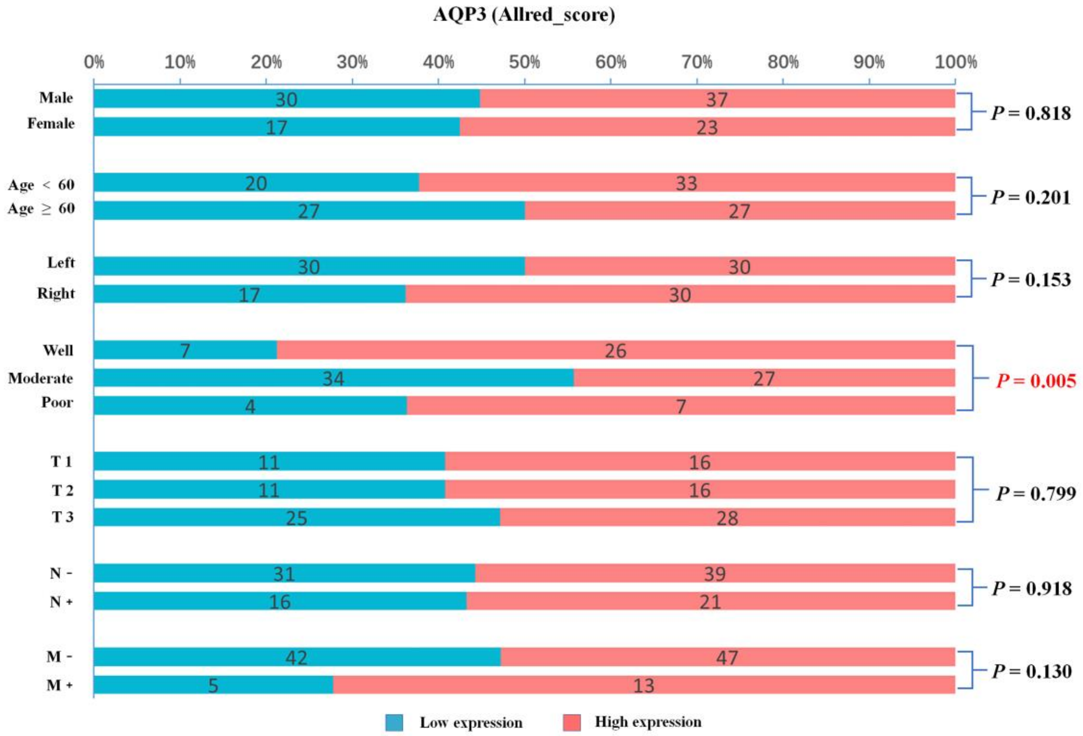


**Fig. S 1** Percentage bar graph about the correlation between AQP3 (Allred_score) of colorectal carcinoma and clinicopathological characteristics. The AQP3 expression was significantly correlated with the degree of differentiation of colorectal carcinoma ($\chi^2$=10.773, *P*=0.005).

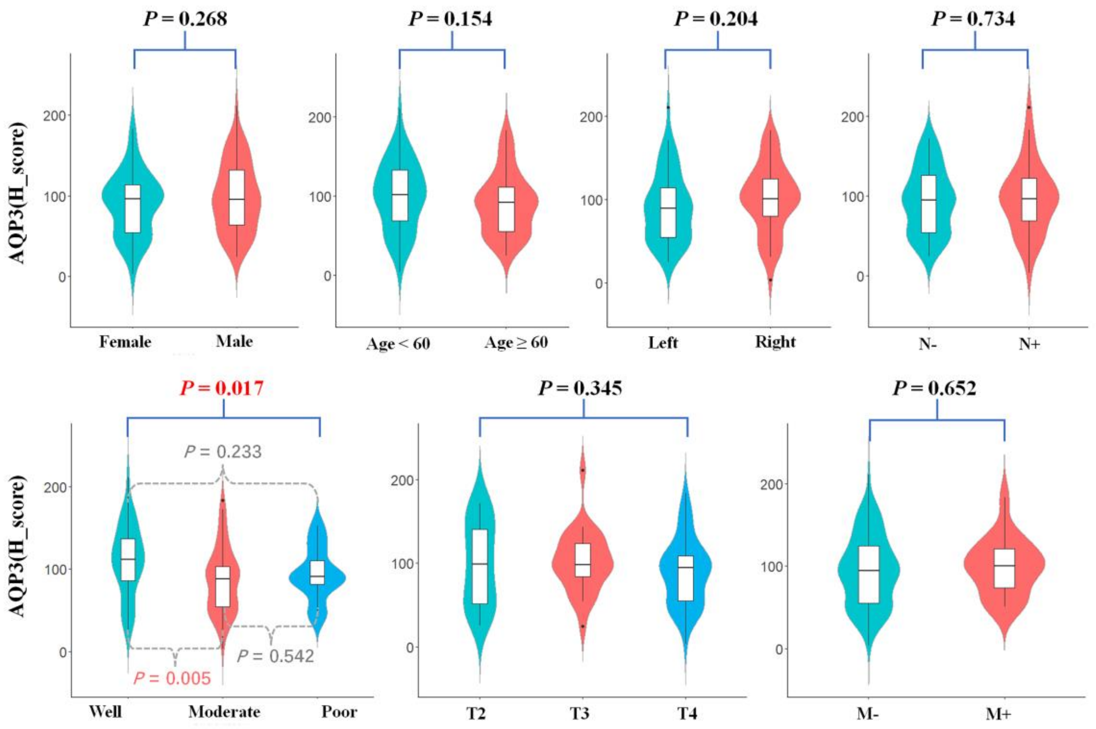


**Fig. S 2** Violin plots about the correlation between AQP3 (H_score) of colorectal carcinoma and clinicopathological characteristics. AQP3 expression was significantly correlated with the degree of differentiation ($F$=4.212, $P$=0.017). In the multiple comparisons of the degree of differentiation between groups, AQP3 expression in well differentiation of colorectal carcinoma was significantly higher than that in moderate differentiation of colorectal carcinoma ($P$=0.005), while there was no significant difference between the other two groups ($P$>0.05).

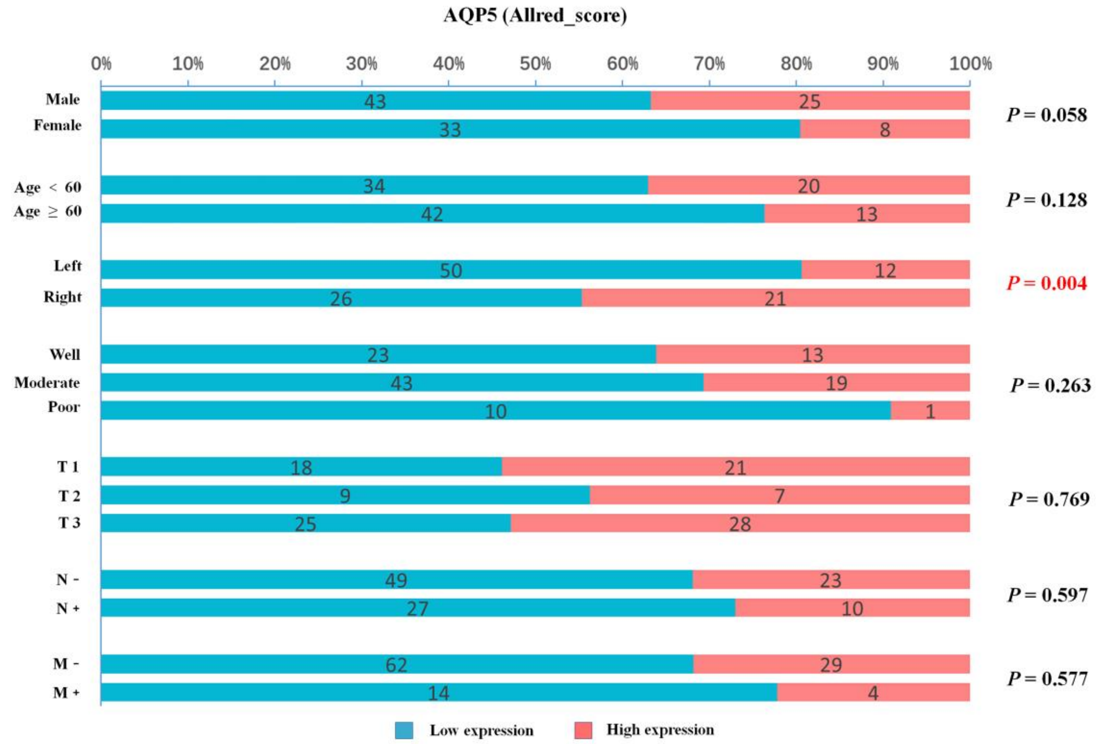


**Fig. S 3** Percentage bar graph about the correlation between AQP5 (Allred_score) of colorectal carcinoma and clinicopathological characteristics. The AQP5 expression was significantly correlated with the location of colorectal carcinoma ($\chi^2$=8.123, $P$=0.004).

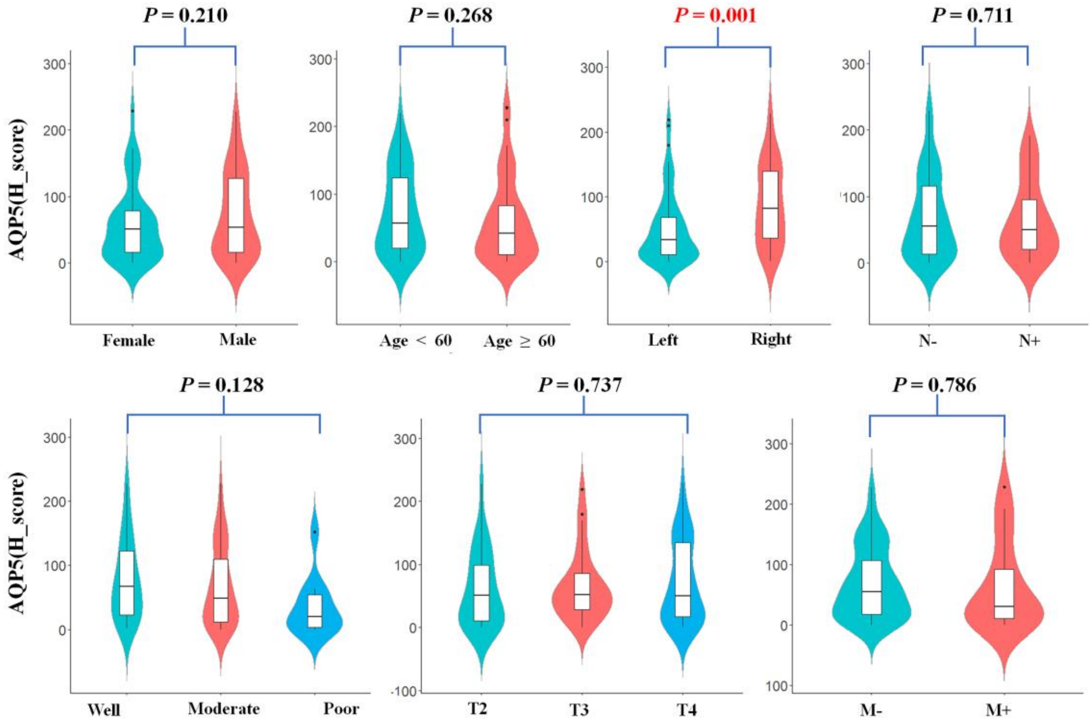


**Fig. S 4** Violin plots about the correlation between AQP5 (H_score) of colorectal carcinoma and clinicopathological characteristics. The AQP5(H_score) expression was significantly lower in the left colon than in the right colon (50.473±54.175 vs 90.052±62.624, $t$=-3.531, $P$=0.001).

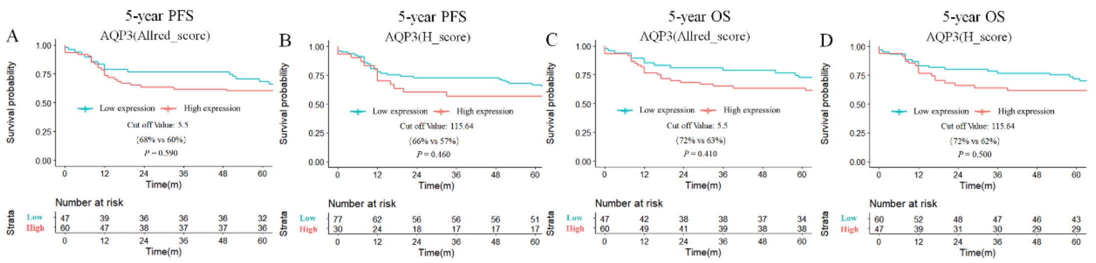


**Fig. S 5** Kaplan-Meier survival curves of AQP3(Allred_score and H_score). For 5-year PFS and OS, there was no significant difference between the high expression group and low expression group of AQP3 in colorectal carcinoma (*P*>0.05).

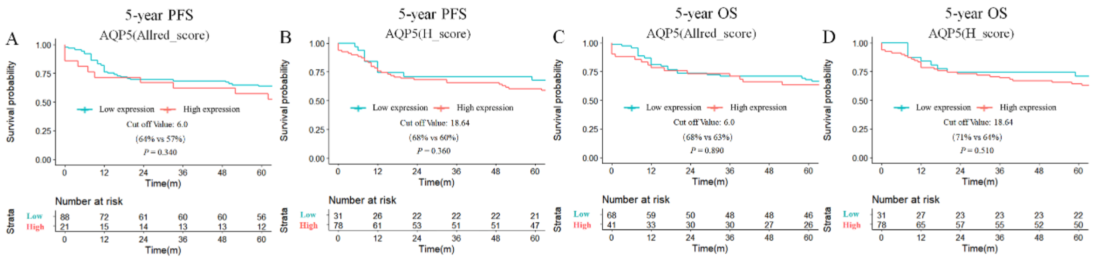


**Fig. S 6** Kaplan-Meier survival curves of AQP5(Allred_score and H_score). For 5-year PFS and OS, there was no significant difference between the high expression group and low expression group of AQP5 in colorectal carcinoma (*P*>0.05).
